# Transcriptomic Analysis and the Effect of Maturity Stage on Fruit Quality Reveal the Importance of the L-Galactose Pathway in the Ascorbate Biosynthesis of Hardy Kiwifruit (*Actinidia arguta*)

**DOI:** 10.3390/ijms23126816

**Published:** 2022-06-19

**Authors:** Diya Lei, Yuanxiu Lin, Qiyang Chen, Bing Zhao, Honglan Tang, Yunting Zhang, Qing Chen, Yan Wang, Mengyao Li, Wen He, Ya Luo, Xiaorong Wang, Haoru Tang, Yong Zhang

**Affiliations:** 1 College of Horticulture, Sichuan Agricultural University, Chengdu 611130, China; 20152539@stu.sicau.edu.cn (D.L.); linyx@sicau.edu.cn (Y.L.); zhaobing199892@gmail.com (B.Z.); 2020305037@stu.sicau.edu.cn (H.T.); asyunting@gmail.com (Y.Z.); supnovel@gmail.com (Q.C.); wangyanwxy@163.com (Y.W.); limy@sicau.edu.cn (M.L.); hewen0724@gmail.com (W.H.); luoya945@163.com (Y.L.); wangxr@sicau.edu.cn (X.W.); htang@sicau.edu.cn (H.T.); 2 Institute of Pomology & Olericulture, Sichuan Agricultural University, Chengdu 611130, China; 3 School of Life Sciences and Engineering, Southwest University of Science and Technology, Mianyang 621010, China; chenqiyang@swust.edu.cn

**Keywords:** hardy kiwifruit (*Actinidia arguta*), fruit quality, ascorbic acid, RNAseq

## Abstract

Hardy kiwifruit (*Actinidia arguta*) has recently become popular in fresh markets due to its edible skin and rich nutritional value. In the present study, different harvest stages of two *A. arguta* cultivars, ‘Issai’ and ‘Ananasnaya’ (“Ana”), were chosen for investigating the effects of maturity on the quality of the fruit. Interestingly, Issai contained 3.34 folds higher ascorbic acid (AsA) content than Ana. The HPLC method was used to determine the AsA content of the two varieties and revealed that Issai had the higher content of AsA and DHA. Moreover, RNA sequencing (RNAseq) of the transcriptome-based expression analysis showed that 30 differential genes for ascorbate metabolic pathways were screened in Issai compared to Ana, which had 16 genes down-regulated and 14 genes up-regulated, while compared to the up-regulation of 8 transcripts encoding the key enzymes involved in the L-galactose biosynthesis pathway. Our results suggested that AsA was synthesized mainly through the L-galactose pathway in hardy kiwifruit.

## 1. Introduction

Hardy kiwifruits (*Actinidia arguta*) have smooth, edible skins and are widely distributed wild germplasm resources in China [1]. Additionally, hardy kiwifruit is distinguished by high frost resistance (up to −30 °C in the dormant period) and may be commercially cultivated in countries with cooler climates [2]. The kiwifruit is a typical respiratory climacteric fruit. Its ripening stage is divided into two stages, morphological ripening and physiological ripening, which are mainly manifested as increased soluble sugar content, decreased hardness, gradually softened taste, gradually decreased total acid and tannin content, and correspondingly decreased acidity of the fruit [3,4]. The harvesting date of hardy kiwifruit is difficult to determine directly by appearance. This is because once the fruit enters the morphologically mature stage, it will be transferred to physiological maturity within a very short period of time [5]. Therefore, mastering the dynamic changes in the quality of the harvested plants will help us harvest at the right time and reduce the losses caused by unacceptable harvesting or late harvesting.

Ascorbic acid (AsA), also known as vitamin C, is an important antioxidant that has a variety of biological effects, such as photosynthesis, defense mechanisms, and cell division [6]. Although AsA is an indispensable substance for human beings, it is completely derived from dietary supplements [7]. More than 90% AsA is derived from fruits and vegetables; thus, ASA content acts as an important factor to evaluate fruit quality [8]. Therefore, it is particularly important to study the anabolism of AsA in plants. There are four ways proposed for the synthesis of AsA in plants, including the L-galactose pathway, the L-gulose pathway, the D-galacturonate pathway, and the myo-inositol pathway [9]. The L-galactose pathway consists of a series of successive reactions, starting from D-mannose-1-phosphate, GDP-L-galactose and L-galactone-1,4-lactone intermediate to the formation of AsA. In addition, AsA can be oxidized to monodehydroascorbate (MDHA) by ascorbate peroxidase (APX) and ascorbate oxidase (AO), then some MDHA can be further reduced by monodehydroascorbate reductase (MDHAR) to form AsA. On the other hand, MDHA can be disproportionated to dehydroascorbate (DHA) and AsA, and DHA can also be reduced to AsA by dehydroascorbate reductase (DHAR) [10]. At present, although there are in-depth studies on AsA and their regulatory mechanisms in fruit, the AsA biosynthetic pathway among different fruit species, developmental stages, and fruit organs is still unclear.

Kiwifruit (*Actinidia* Lindl.) is the most economically important of fruit trees and is favored by consumers owing to its high vitamin C content [11]. Many studies have shown that biosynthesis is the main cause of AsA accumulation in kiwifruit fruit, and the AsA content in fruit development is regulated by both synthesis and regeneration [12]. Moreover, there was a significant difference in the AsA content among different kiwifruits, and there was wide variation in AsA content in most cultivars of A. argute fruit; the AsA content was much higher than that of *A. deliciosa* and *A. chinensis* [13]. For understanding the AsA accumulation among different genotypes, two different hardy kiwifruit cultivars were selected to perform changes in the AsA content and to reveal the molecular regulation of AsA accumulation in the fruit by transcriptome analysis. The results would be beneficial for kiwifruit breeding strategies, with the purpose of increasing AsA content.

## 2. Results

### 2.1. Effect of Maturity Stage at Harvest on Changes in Phenotypic Characterization

**Figure 1:**
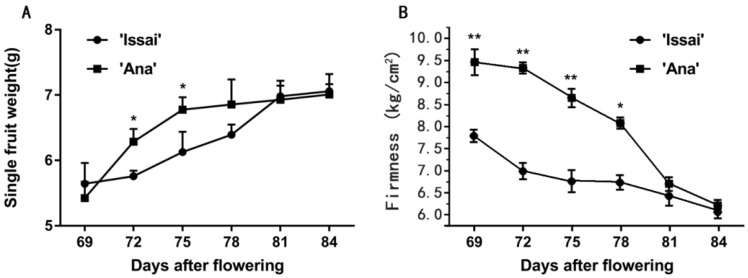
The effect of maturity stage at harvest on changes in fresh weight (**A**) and flesh firmness (**B**). Data are presented as mean ± standard error from three biological replicate assays (** *p* < 0.01, * *p* < 0.05). The error bars represent standard error of the means.

The fresh weight of two cultivars rapidly increased during early harvest stages (69-81DAF), slowly increased during 81–83 DAF, and the fresh weight of Ana was significantly higher than Issai (Figure 1A). The flesh firmness of two kiwi berries decreased gradually and varied in the range of 9.46–6.23 kg/cm^2^ and 7.8–6.1 kg/cm^2^ during the harvest period, respectively, with no significant difference between them at 84DAF (Figure 1B).

**Figure 2:**
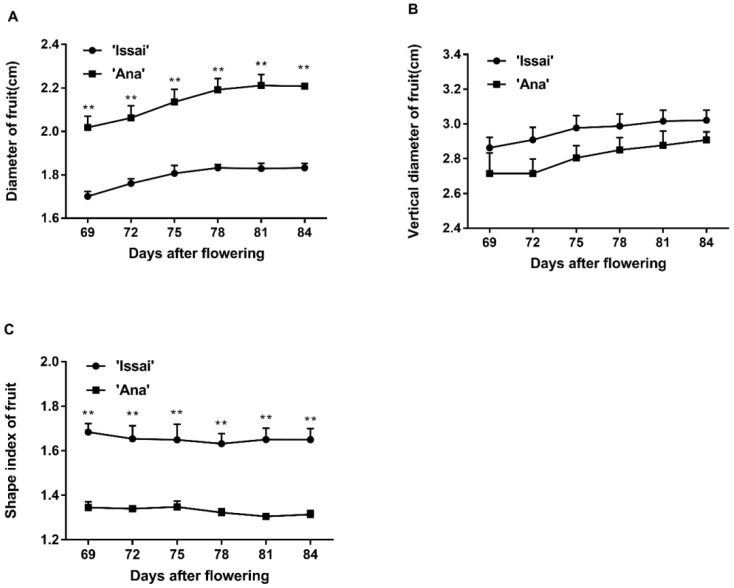
The effect of maturity stage at harvest on changes in diameter (**A**), vertical diameter (**B**), and shape index (**C**) of the fruit. Data are presented as mean ± standard error from three biological replicate assays (** *p* < 0.01). The error bars represent standard error of the means.

As shown in Figure 2, the growth of diameter and vertical of the fruit increased gradually during harvest period, while the shape index of both varieties has a slight decrease. For the two cultivars, the fruit diameter of Ana was significantly higher than that of Issai (Figure 2A); however, the vertical diameter of Ana was lower than that of Issai (Figure 2B). The fruit shape index of Ana was significantly lower than Issai, which indicated that the fruit shape between the two cultivars was different (Figure 2C).

### 2.2. The Effect of Maturity Stage at Harvest on Changes in the Nutritional Quality of the Fruit

**Figure 3:**
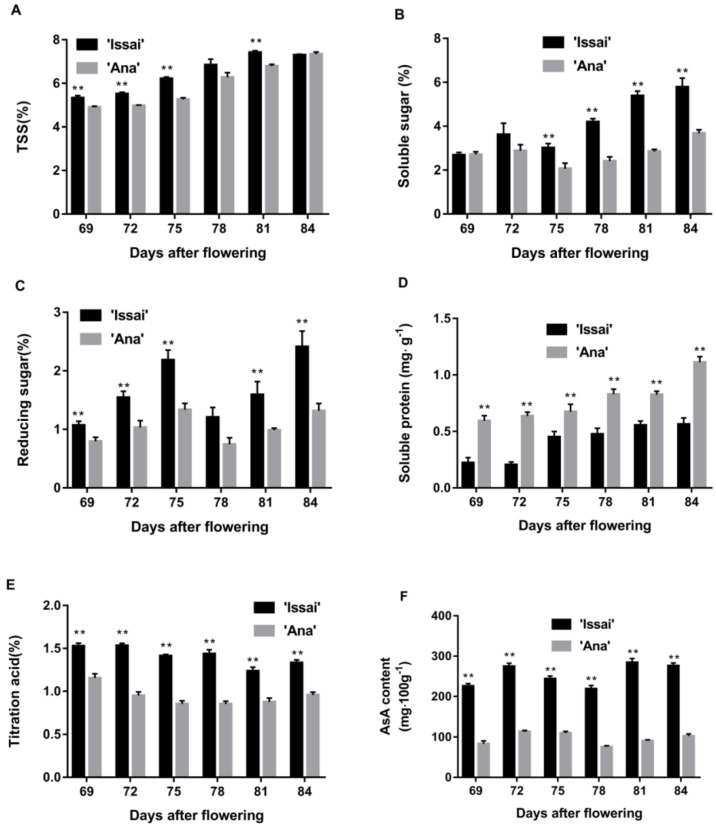
The effect of maturity stage at harvest on changes in TSS (**A**), soluble sugar (**B**), reducing sugar (**C**), soluble protein (**D**), titration acid (**E**), and AsA content (**F**). Data are presented as mean ± standard error from three biological replicate assays (** *p* < 0.01). The error bars represent standard error of the means.

Changes in the nutritional quality of the fruit at different harvest stages are shown in Figure 3. Obviously, Issai was significantly higher than Ana on five nutritional quality indexes, such as TSS, reducing sugar, TA, soluble sugar, and AsA content (Figure 3A–C,E,F), while soluble protein content was on the contrary (Figure 3D). Abnormally, there was no significant difference on TSS at 84 DAF, reducing sugar at 78 DAF, or soluble sugar at 69 DAF and 72 DAF. Further, the content of TSS and soluble protein in both varieties showed a gradually increasing trend from 69 DAF to 84DAF, while reducing sugar, soluble sugar, and AsA contents showed a fluctuated variation. Overall, the TA content of Issai and Ana declined slightly from 69 DAF to 78 DAF, and a slight increasing trend was observed at 84 DAF (Figure 3E). In addition, Issai had significantly higher AsA content than Ana at six harvest times (Figure 3F).

### 2.3. Comparison of ASA and DHA Content

**Figure 4:**
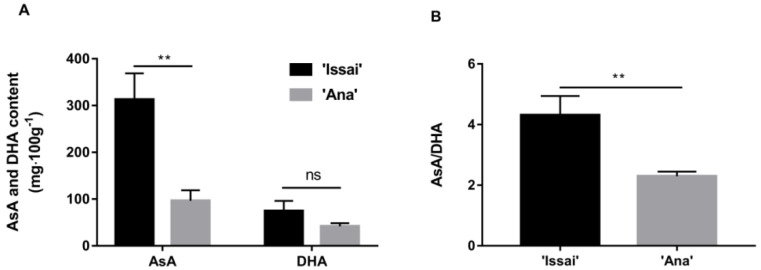
The content of AsA and DHA (**A**), and AsA/DHA ratio (**B**) at 84 DAF. The data are presented as mean ± standard error from three biological replicate assays (** *p* < 0.01). The error bars represent standard error of the means.

Because of the large difference in AsA content between Issai and Ana, the HPLC method was used to determine the AsA content of the two varieties at 84 DAF. HPLC chromatogram traces of AsA and total AsA are shown in Supplementary Figure S1. The HPLC chromatogram profiles showed a clear separation of the peaks at a variable UV wavelength, which demonstrated that the operating parameters of HPLC methodology adopted in the presented study are able to satisfactorily separate ascorbic acid. The HPLC analysis revealed that the Issai had the higher content of AsA and DHA. Respectively, the AsA content in Issai was 302.12 mg/100 g, which was 3.34 times that of Ana. Although there was no significant difference in DHA content between the two varieties, the DHA content of Issai was 1.78 times that of Ana (Figure 4A). In the case of the AsA/DHA ratio, Issai was significantly higher than Ana (Figure 4B).

### 2.4. Activities of Key Enzymes Involved in AsA Metabolism

**Figure 5:**
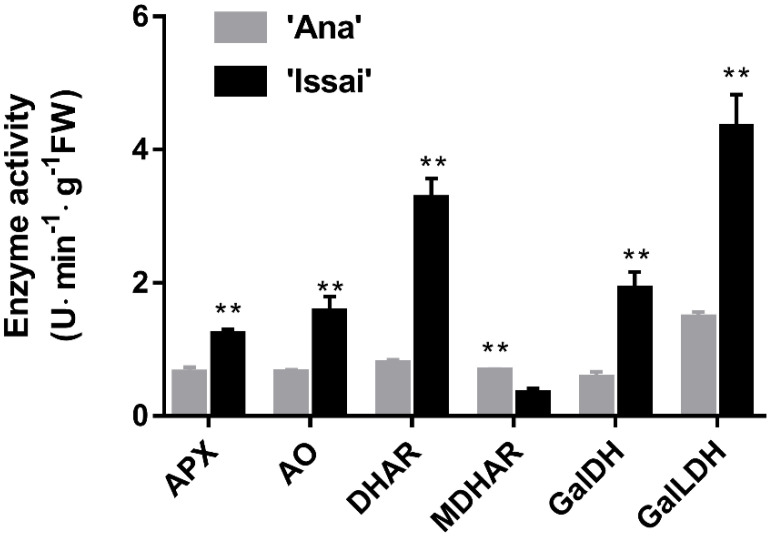
Enzymatic activity of ascorbic acid biosynthesis pathway at 84 DAF. Data are presented as mean ± standard error from three biological replicate assays (** *p* < 0.01). The error bars represent standard error of the means.

The activity of AsA metabolism–related enzymes in two hardy kiwifruits at 84 DAF were shown in Figure 5. The overall enzymatic activity of Issai was significantly higher than that of Ana; however, the MDHAR activity of Ana was 2.09 times higher than Issai. To the best of our knowledge, GalLDH is the key enzyme in the L-galactose pathway. In Issai, the activity of the GalLDH enzyme was the highest level and was 2.97 times as much as Anna. The activity of the DHAR enzyme in Issai was 3.24 U/min·g FW, which was significantly higher than that of Ana. The APX, AO, and GalDH enzyme activities of Issai were 1.94, 2.44, and 3.45 times higher, respectively, than those of Ana (Figure 5).

### 2.5. DEGs Obtained and qRT-PCR Validation

In order to further explore the molecular mechanism of the two *A. arguta* varieties of the AsA metabolism, RNAseq was carried out using the fruit at 84 DAF of Ana and Issai. The total cDNA library was prepared from the fruit of the two cultivars, repeated three times, and sequenced using an Illumina double-sequencing platform, resulting in 55,686,882, 53,457,420, and 56,151,694 raw reads in Issai and 57,088,922, 5,088,658, and 58,250,288 raw reads in Ana. After cleaning and quality checking, 53,933,324, 51,733,194, 54,239,872, 55,014,194, 48,602,224, and 56,524,894 clean reads were generated from the two cultivars’ cDNA libraries, respectively (Supplementary Table S2). The percentage of Q20 between Issai and Ana is greater than 96%, the percentage of Q30 is greater than 91%, and the error rate of the 6 samples is 0.02%. The GC content between Issai and Ana was consistent, with Issai at 46.8% and Ana at 47% (Supplementary Table S2). This shows that the sequencing data can meet the requirements of subsequent analysis.

Through the analysis of transcriptome sequencing results, 30 differential genes for ascorbate metabolic pathways were screened, including the key gene for the synthesis pathway of L-galactose metabolism: GME, GGP, GPP, GalDH, and GalLDH; MIOX in the inositol synthesis pathway; and ALDH, UGDH, GLCAK, etc.; which were the key genes for the regeneration pathway of AsA, MDHAR, AO, and APX. Among the 30 differentially expressed genes, 16 genes were down-regulated and 14 genes were up-regulated. We have chosen 14 expressive genes to perform fluorescent quantitative PCR (qPCR) verification. From the results of qPCR, 9 genes were up-regulated and 5 genes were down-regulated (Supplementary Figure S2), which is consistent with the sequencing results of the transcriptome. This shows that the results of the transcriptome of the hardy kiwifruit are accurate and reliable.

### 2.6. Expression Patterns of AsA Metabolism Genes

**Figure 6:**
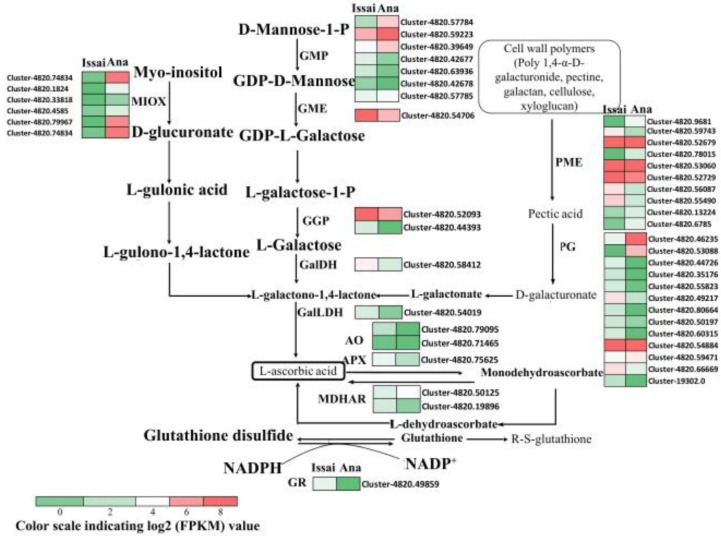
Schematic of AsA metabolism pathway in Ana and Issai. Expression patterns of DEGs are represented by log2 FPKM value and shown as a heatmap at the side of each step. The cells of the heatmap from left to right represent the expression level in Issai and Ana, respectively. The full name of the abbreviated genes is listed in the Table S3.

To explore in depth the molecular difference in AsA metabolism between Issai and Ana, the expression profiling of transcripts involved in AsA biosynthesis was analyzed based on the transcriptome data. The results (Figure 6, Supplementary Table S3) showed that several transcripts-encoding enzymes involved in the predominant AsA biosynthetic pathway (L-galactose pathway) were largely up-regulated in Issai. For instance, the transcript Cluster-4820.52093 and Cluster-4820.44393, both encoding GGP, expressed 2.6 and 4.6 folds higher, respectively, in Issai than in Ana. The other key enzymes in this pathway including GMP, GME, GalDH, and GalLDH were also significantly up-regulated in Issai. Moreover, the expression levels of transcripts encoding pectinesterase (PME) and polygalacturonase (PG) involved in the early steps of D-galacturonate biosynthesis pathway were also significantly enhanced in Issai. Our results suggested a higher flux of AsA biosynthesis in Issai than in Ana. However, notably, the expression of transcripts encoding MIOX was significantly repressed in Issai. In addition, the transcripts encoding AO, APX, and glutathione reductase (GR) involved in the AsA-GSH cycle were also up-regulated in Issai. Two transcripts encoding MDHAR were found to be up- and down-regulated in Issai, respectively. The FPKM values of transcripts involved in the AsA metabolism pathway are shown in Supplementary Table S3.

## 3. Discussion

The early and late fruit harvesting period is closely related to fruit yield, quality, and storability, and high maturity usually couples with poor storage resistance. When maturity is low, fruit quality, size, and flavor are not up to the requirements, resulting in poor quality. Grasping the dynamic changes in the physiology and biochemistry indexes of kiwifruit from its mature stage to its physiological maturity is the key to determine the best harvest time for kiwifruit. In our study, at 78 DAF, the hardy kiwifruit had basically the same size and volume, indicating that the morphological maturity had been completed at this time. Although there were morphological differences in the two varieties of hardy kiwifruit, there was no difference in weight for a single fruit. Soluble solids contain many components that can be solubilized in water, such as sugar, acids, vitamins, and some minerals. It is an important to evaluate the quality of fruits and vegetables as an integrated index [14]. Currently, soluble solids are used as harvest indicators for kiwifruit. The harvest maturity of *A. deliciosa* ‘Hayward’ revealed a soluble solids content of 6.2% at the minimum harvest time [15]. From our results, the minimum harvest time can be met after three days of morphological maturity. The soluble sugar and reducing sugar content of hardy kiwifruit showed an increasing trend during the harvest period. During physiological maturity, the sugar was mainly converted to sucrose.

AsA is not only an essential nutrient for humans but also an antioxidant for scavenging reactive-oxygen species (ROS) in plants. Kiwifruit is rich in AsA—one of the important measures of the fresh quality. Several studies have proposed that there are certain differences in ASA content among different varieties of Kiwifruit, with Issai an AsA-rich cultivar [16]. In the present study, our results showed that the AsA content in Issai was 3.34 folds higher than in Ana, and a higher ratio of AsA/DHA was found in Issai, indicating that Issai has a higher AsA turnover and regeneration capability [17]. To explore in depth the molecular reason for this difference, the RNAseq-based expression profiles of AsA biosynthesis transcripts were estimated. As is generally known, the L-galactose and D-galacturonate pathway was proposed as the predominant and alternative pathway for AsA biosynthesis [18], and changes in the expression of genes in both pathways could manipulate AsA accumulation. GalDH and GalLDH were considered to be the key enzymes for the synthesis of AsA via the L-galactose pathway [19]. GalDH uses NAD+ as an electron acceptor to oxidize L-galactose-1,4-lactone and catalyze the oxidation of the final precursor L-galactose-1,4-lactone to AsA [20]. In Arabidopsis thaliana, inhibition of the GalDH and GalLDH gene resulted in an approximately 50% reduction in AsA content [21]. Based on our present transcriptome data, we found that the expression levels of transcripts encoding enzymes involved in the L-galactose biosynthesis pathway were significantly up-regulated in Issai, including GME, GGP, GalDH, and GalLDH. These results suggested that AsA was synthesized mainly through the L-galactose pathway in hardy kiwifruit, which is consistent with the previous reports in Arabidopsis and Tomato [22,23]. Overexpression of the kiwifruit GGP gene, a homolog in tobacco, results in a threefold increase in AsA content, and thereby GGP has been suggested as a rate-limiting route for AsA biosynthesis [24,25]. In our results, the transcript level of GGP (Cluster-4820.44393) in Issai is about 5 folds higher than in Ana, indicating that GGP is the key gene for the AsA accumulation in hardy kiwifruit. Moreover, the increase in PME and PG expression in the alternative D-galacturonate pathway could lead to an increased AsA production in Solanaceae [26]. Our results also showed that the expression levels of the transcripts encoding the enzyme PME and PG in the alternative D-galacturonate pathway significantly increased in Issai, which might contribute to the higher AsA content in Issai, indicating that the alternative D-galacturonate pathway is also important in hardy kiwifruit AsA biosynthesis. APX, AO, GR, DHAR, and MDHAR are key enzymes in the oxidation and recycling of AsA and play a very important role in regulating the content of ASA and DHA. Ishikawa and Shigeoka [27] conducted an APX study and found that APX activity decreased as the concentration of AsA decreased. AO maintains the redox ratio of the AsA pool mainly by regulating the AsA redox state in vitro of plant cell protoplasts. In the present study, our results found that APX and AO were higher in Issai fruits with high concentrations of AsA, suggesting that APX and AO are closely related to AsA content. The expression and enzyme activities of MDHAR were both low in the two cultivars. At the same time, the enzyme activity in the high AsA content cultivar was lower than that in the low AsA content cultivar, indicating that MDHAR played a minor role in the metabolic pathway of AsA in hardy kiwifruit. This is consistent with the findings of Li [28]. DHAR is an important enzyme for the regeneration of AsA, which can contribute to the accumulation of AsA in ‘white’ kiwifruit [29]. Chen [30] overexpressed the wheat DHAR in tobacco, resulting in an increase in the AsA/DHA ratio and a fourfold increase in AsA content. Our results found that although the difference in DHAR gene is not significant, the enzyme activity differs by more than 4 times. What’s more, although the oxidation and recycling of AsA in Issai is more active than that in Ana because of the higher expression of oxidation and recycling transcripts in Issai, the expression of all oxidation and recycling transcripts is relatively lower than the biosynthetic transcripts, indicating that the AsA accumulation is mainly from biosynthesis other than from recycling. By contrast, the expression of MIOX family genes in another alternative AsA biosynthesis pathway is lower in Issai than that in Ana, indicating that MIOX might not have obvious effects on AsA accumulation in hardy kiwifruit, while it might including other functions such as construction of cell wall and the biosynthesis of sugar alcohol as previously suggested [31].

## 4. Materials and Methods

### 4.1. Plant Materials and Sample Preparation

The plant materials consisted of two cultivars of 4-year-old hardy kiwifruit, *A. arguta* cv. Issai and *Actinidia arguta* cv. Ananasnaya (“Ana”), which were grown in Ya’an, Sichuan province, China. When the total soluble solid content (TSS) had reached about 5%, the fruits were collected at 69 DAF (days after flowering), 72DAF, 75 DAF, 78 DAF, 81 DAF, and 84 DAF. For each variety, 90 fruits were harvested without physical injuries, along the canopy, and immediately transported to a laboratory in Sichuan Agricultural University. Each group of sixty fruits was kept in a clean plastic box in a refrigerator at 4 °C and another thirty fruits were rapidly frozen in liquid nitrogen and stored at −80 °C.

### 4.2. Determination of Fruit Quality

Thirty fruits were selected randomly to measure appearance qualities without injuries, the single fruit weight(g) by electronic balance, the transverse and longitudinal diameters of the fruit(cm) by vernier caliper, and the firmness by a hardness meter (DWGY-4) with a 10 mm plunger. TSS was measured using a hand-held refractometer (PAL-1, Atago, Japan) by dropping a sample of the fruit juice on it and was expressed as “%”. The content of soluble sugar (%) was obtained by using anthrone colorimetry [32], the titratable acid (TA) content (%) by using acid-base titration [33], the AsA content (mg/100g) by using 2,6-Dichlorononylphenol titration [34], the content of soluble protein (mg/g) by using the Coomassie brilliant blue method [35], and the content of reducing sugar by using the 3,5-dinitrosalicylic acid colorimetric method [36].

### 4.3. HPLC Analysis of AsA

AsA level was evaluated using high-performance liquid chromatography (HPLC). For each variety, 180 fruits were mixed with all six periods. AsA and the total AsA level were determined essentially as described by Huang [37] and Gökmen [38], respectively. Dehydroascorbic acid (DHA) content was evaluated as the difference between total and reduced AsA levels.

### 4.4. Measurement of AsA Metabolism–Related Enzymes

For the related enzymes: the L-Galactono-1,4-lactone dehydrogenase (GalLDH) was determined according to Tabata [39], the L-galactose dehydrogenase (GalDH) was measured with the reference to Gatzek [21], and the four enzymes, L-ascorbate oxidase (AO), L-ascorbate peroxide (APX), dehydroascorbate reductase (DHAR), and monodehydroascorbate reductase (MDHAR)were determined by spectrophotometry [40]. All samples were extracted and analyzed in triplicate for every index above.

### 4.5. RNA-seq and Selection of Genes Involved in the AsA Matabolism

The total RNA was isolated separately from fruits following a CTAB–LiCl precipitation method [41]. cDNA was synthesized from 1 μg of total RNA using PowerScript III reverse transcriptase (Invitrogen) according to the suppliers’ manual. Sequencing libraries were generated using an RNA Library Prep Kit for Illumina (NEB, Ipswich, MA, USA). The library preparations were sequenced on a HiSeq X Ten platform (Illumina, San Diego, CA, USA) and paired-end reads were generated. In total, 6 libraries were sequenced for the two cultivars with 3 biological repetitions. The genes involved in AsA metabolism were identified from the whole-genome sequences of *Actinidia chinensis* var. *chinensis* (accession number: NKQK00000000.1).

### 4.6. qRT-PCR Analysis

Fourteen novel transcripts related to AsA metabolic pathways—including GDP-D-mannose 3’,5’-epimerase (GME), GDP-L-galactose phosphorylase (GGP), L-galactose 1-phosphate phosphatase (GPP), GalDH, GalLDH, glucuronidase (GLCAK), myo-inositol oxygenase (MIOX), UDP-glucose 6-dehydrogenase (UGDH1, 2), aldehyde dehydrogenase (ALDH), AO, APX1, APX2, and MDHAR—were monitored via qPCR using the 2× SYBR Green mixture (TaKaRa, Dalian, China) and conducted on a CFX96 Real-Time PCR Detection System (Bio-Rad, Hercules, CA, USA). PCR primers designed were used Beacon Design 7.9, primers Biotechnology (Shanghai) Co., Ltd. synthesis (Supplementary Table S1). Using cDNA as a template and the *β-actin* gene as an internal reference, synthetic primers were used for qRT-PCR amplification to detect the transcription level of related genes.

The qPCR protocol was as follows: 95 °C for 30 s, followed by 40 cycles at 95 °C for 5 s, 55 °C for 30 s, and 72 °C for 30 s. At the end of each experiment, a melt curve analysis was conducted at 72 °C for 5 s. Three biological replicates were made for each template, and the mean value was taken as the CT value of the corresponding gene of interest. The ΔΔCT method was used to process the fluorescence quantitative PCR amplification data, and the relative expression amount of the target gene was expressed by the 2^−ΔΔCT^ value. The sofeware-PermutMatrix-1.9.3 was used for cluster analysis.

### 4.7. Statistical Analysis

Data were expressed as mean values ± SD and were subjected to one-way analysis of ANOVA using SPSS 19.0 software (SPSS Inc., Chicago, IL, USA). The differences at *p* ≤ 0.05 were considered as significant.

## 5. Conclusions

In the present study, at 78 DAF, both of the hardy kiwifruit varieties reached morphological maturity. At the same time, combined with the content of TSS, we argue that the best harvest period of hardy kiwifruit is about 3 days after morphological maturity. The AsA content in Issai was 3.34 folds higher than in Ana, and a higher ratio of AsA/DHA was found in Issai, indicating that Issai has a higher AsA turnover and regeneration capability. Based on transcriptome data, we suggested that AsA was synthesized mainly through the L-galactose pathway in hardy kiwifruit.

## Data Availability

Not applicable.

## Supplementary Materials

The following supporting information can be downloaded at: https://www.mdpi.com/article/10.3390/ijms23126816/s1.
