# Supplementary material for: Transcriptomic Analysis and the Effect of Maturity Stage on Fruit Quality Reveal the Importance of the L-Galactose Pathway in the Ascorbate Biosynthesis of Hardy Kiwifruit (Actinidia arguta)
Source: Int J Mol Sci. 2022 Jun 19;23(12):6816. doi: 10.3390/ijms23126816 (PMC9223753; doi:10.3390/ijms23126816)
Supplement: Supplementary file 1 [file ijms-23-06816-s001.zip › ijms-1738076-supplementary.pdf]

Supplemental Figure S1

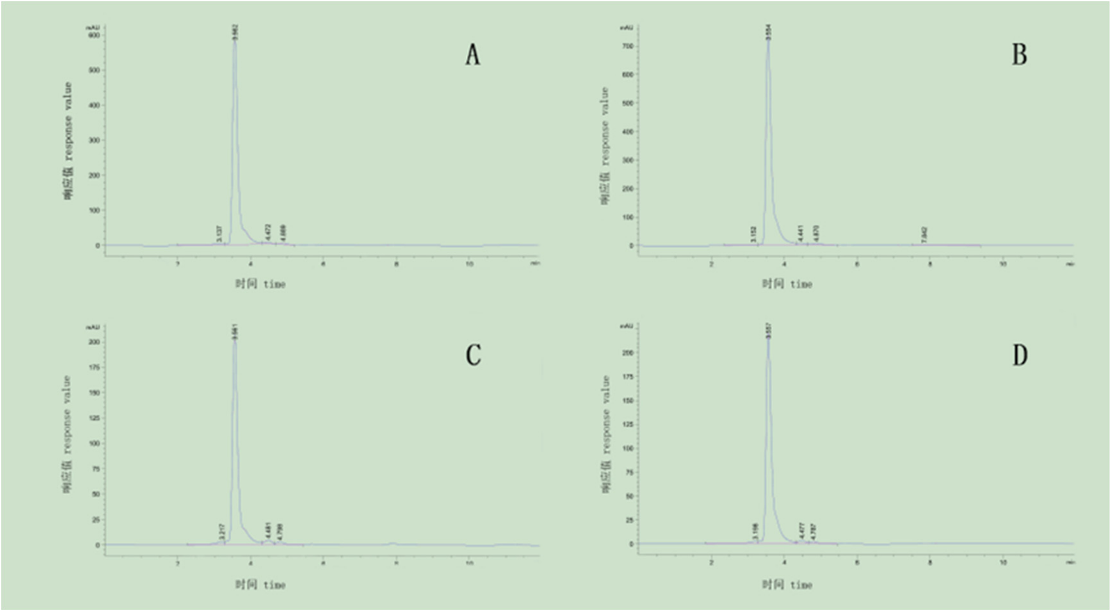

Supplemental Figure S2

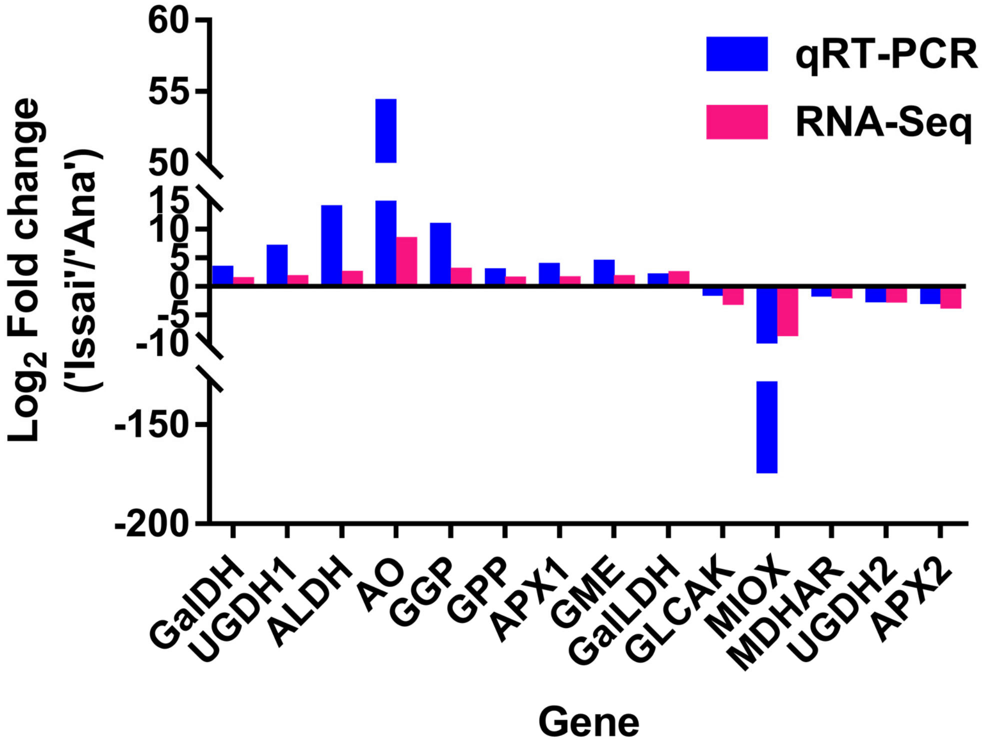

Table S1. Primer sequences of real-time quantitative.

Gene	Gene_id	Enzyme	Sequence of primer(5'--3')	
GME	Cluster-4820.54706	GDP-D-mannose 3', 5'-epimerase	GCTTCATTCAGTCCAATC	AGAACCTCTTAACACCATT
GGP	Cluster-4820.52093	GDP-L-galactose phosphorylase	TTCCAGGCTTATTACTTAG	CACCATTCAGAGTAGTTAT
GPP	Cluster-4820.22737	L-galactose 1-phosphate phosphatase	TGTGACGATTGCTGATAA	ACCATTCTCCTCTCCATA
GalDH	Cluster-4820.58412	L-galactose 1-phosphate phosphatase	GATTGTGAATGAGACGATT	GGAGTCCTGTAATACCAA
GalLDH	Cluster-4820.54019	L-galactono-1,4-lactone dehydrogenase	TCCTATTGACGAGCAAGT	AGCGAGCAAGATAGAAGA
MDHAR	Cluster-4820.50125	monodehydroascorbate reductase (NADH)	TAGAAGCAGACACGATTG	ACCATCAACCTGTATTCC
GLCAK	Cluster-4820.18044	glucuronokinase	ATAGGTTGAATAATGAAGGT	CATACAATAGCACTGGAA
MIOX	Cluster-4820.79967	inositol oxygenase	GCGTCAATCACATTAACC	CTCGTCCACAACATCATT
UGDH1	Cluster-4820.32989	UDPglucose 6-dehydrogenase	CTCTTCCGAGAATTACTG	CATTGCTTCACTACTTCA
UGDH2	Cluster-4820.56325	UDPglucose 6-dehydrogenase	AAGTGGCTGTGGTTGATA	AAGACCTGGCTCATAGATG
ALDH	Cluster-4820.56323	aldehyde dehydrogenase (NAD+)	ATGGAATGACTTCAAGATG	GAGTGACCTGATGAGAAT
AO	Cluster-4820.79095	L-ascorbate oxidase	ACCGAAGGAGTTGTCATT	TGTGAGATGGATGCTGTT
APX1	Cluster-4820.75625	L-ascorbate peroxidase	TTGTTCCGTCCATATTGA	TTATTCTCTGGTCTGCTAAT
APX2	Cluster-4820.20297	L-ascorbate peroxidase	GAAGAGCACATCCAGAGA	TCCACGAAGTATGAATTGTC
Actin			GCTTACAGAGGCACCACTCAACC	CCGGAATCCAGCACAATACCAG

Table S2 Throughput and quality of RNA-Seq of *A.argura*

**Sample**	**Raw Reads**	**Clean reads**	**Clean bases**	**Error(%)**	**Q20(%)**	**Q30(%)**	**GC(%)**
K3_iss_1	55686882	53933324	8.09G	0.02	96.96	92.50	46.79
K3_iss_2	53457420	51733194	7.76G	0.02	97.01	92.62	46.86
K3_iss_3	56151694	54239872	8.14G	0.02	97.10	92.80	46.72
K4_ana_1	57028922	55014194	8.25G	0.02	96.93	92.44	47.54
K4_ana_2	51088658	48602224	7.29G	0.02	96.58	91.72	47.15
K4_ana_3	58250288	56024894	8.4G	0.02	96.74	92.05	47.37

Sample: K3_iss is 'Issai' and K4_ana is ' Ana '.

Supplemental Table S3

Gene name	gene id	K3-1-FPKM	K3-2-FPKM	K3-3-FPKM	K4-ana-FPKM-1	K4-ana-FPKM-2	K4-ana-FPKM-3
GMP	Cluster-4820.57784	3.3	3.05	2.53	33.25	34.77	25.96
	Cluster-4820.59223	45.52	48.77	44.69	167.59	114.93	129.32
	Cluster-4820.39649	18.37	12.91	12.66	26.65	31.41	39.92
	Cluster-4820.42677	7.64	7.45	8.88	1.38	1.69	1.27
	Cluster-4820.63936	5.17	5.33	6.47	0.7	0.8	1.12
	Cluster-4820.42678	1.06	1.24	2.25	0	0.19	0.23
	Cluster-4820.57785	11.68	9.19	9.99	13.17	17.62	10.89
GME	Cluster-4820.54706	139.11	163.34	167.09	37.04	44.6	44.19
GGP	Cluster-4820.52093	496.93	503.72	562.22	42.69	71.44	66.91
	Cluster-4820.44393	7.69	7.33	4.83	0	0	0.52
GalDH	Cluster-4820.58412	17	19.64	22.56	6.96	7.66	5.5
GalLDH	Cluster-4820.54019	9.07	6.69	5.62	0.88	1.31	1.39
PME	Cluster-4820.9681	0.1	0.04	0	13.62	13.98	6.88
	Cluster-4820.20852	2.98	3.03	2.76	0.07	0.08	0
	Cluster-4820.59743	26.73	18.68	17.12	2.58	2.87	3.08
	Cluster-3379.1	0	0	0	4.58	2.64	4.94
	Cluster-4820.52679	1527.47	1427.01	1541.78	4687.66	3529.39	3646.05
	Cluster-4820.78015	0.76	0.11	0.26	4.57	9.19	4.51
	Cluster-3379.0	0	0.06	0	4.29	1.34	3.51
	Cluster-4820.53060	958.89	654.87	721.92	164.19	134.87	255.07
	Cluster-4820.52729	475.68	293.69	368.17	57.65	124.74	82.89
	Cluster-2131.0	0	0	0.04	0.62	2.51	2
	Cluster-4820.56087	22.03	25.11	25.34	4.8	4.03	6.15
	Cluster-4820.14246	1.89	1.21	1.97	0	0	0
	Cluster-4820.14247	1.4	0.73	1.12	0	0	0
	Cluster-4820.85004	1.71	2.49	1.01	0.08	0	0
	Cluster-4820.78016	0	0	0	0.32	0.4	0.53
	Cluster-4820.78938	1.59	1.19	1.38	0.15	0.24	0.21
	Cluster-4820.55490	36.12	22.87	31.09	0	3.78	13.48
	Cluster-19727.0	1.18	1.4	1.49	0	0.09	0
	Cluster-4820.13224	2.46	2	1.88	8.01	9.1	4.66
	Cluster-4820.6785	0.64	1	1.29	4.87	16.35	6.67
	Cluster-4820.75011	0.49	1.58	1.51	0	0	0
	Cluster-4820.78939	1.24	1.99	2.41	0	0	0
PG	Cluster-4820.46235	15.96	9.33	5.6	2516.92	2198.37	2015.48

	Cluster-4820.53088	0.17	0	0	40.33	35.06	47.26
	Cluster-4820.44726	6.98	8.37	8.24	0.15	0.27	0.13
	Cluster-4820.35176	4.96	4.49	5.69	0.06	0	0.5
	Cluster-4820.75059	2.68	1.72	1.94	0	0	0
	Cluster-4820.55823	7.99	5.91	5.79	0.76	0.54	0.62
	Cluster-4820.49217	23.79	22.32	25.19	3.56	6.11	2.97
	Cluster-4820.35171	2.98	0.73	2.25	0	0	0
	Cluster-4820.80664	7.99	3.04	4.94	0.33	0.16	0.28
	Cluster-4820.20235	2.42	2.17	1.69	0.12	0	0
	Cluster-4820.82064	0	0	0	0.48	3.67	2.12
	Cluster-4820.31576	0	0	0.01	1.25	0.2	0.52
	Cluster-4820.50197	6.35	5.49	4.61	1.3	2.19	1.46
	Cluster-4820.67482	1.3	0.93	0.72	0.09	0.11	0
	Cluster-4820.60315	7.6	6.99	3.98	0.42	0.44	0.22
	Cluster-4820.54884	703.2	484.66	530.05	3487.01	2607.84	2160.49
	Cluster-4820.57605	4.11	4.28	3.1	0.22	0.44	1.59
	Cluster-4820.57606	2.06	1.85	2.35	0.18	0.95	0
	Cluster-4820.59471	15.59	13.8	11.7	17.62	21.9	22.91
	Cluster-19302.1	1.42	0.26	2.07	0	0	0
	Cluster-20704.2	1.13	1.4	0.81	0.21	0	0
	Cluster-4820.66669	25.66	22.82	27.14	8.55	9.79	7.89
	Cluster-12103.0	0.51	0.49	2.02	0	0	0
	Cluster-20704.0	0.54	0.29	1.21	0	0.11	0
	Cluster-19302.0	2.62	1.84	7.06	0	0.16	0.41
MDHAR	Cluster-4820.50125	9.4	8.67	6.71	20.13	14.4	11.23
	Cluster-4820.19896	5.74	6.99	6.46	0.84	1.6	1.53
AO	Cluster-4820.79095	3.01	4.05	4.8	0.03	0	0
	Cluster-4820.71465	0.61	0.66	0.76	0	0	0.07
APX	Cluster-4820.20297	0.07	0.07	0.42	1.28	1.54	0.86
	Cluster-4820.75625	9.04	13.24	10.9	2.87	3.94	3.76
GR	Cluster-4820.49859	10.15	11.39	9.14	0.22	0	0
	Cluster-4820.51676	161.87	146.71	142.55	273.27	227.07	229.37
MIOX	Cluster-4820.74834	0.59	0.61	1.25	85.43	91.47	129.61
	Cluster-4820.1824	0.14	0.15	0	6.05	8.24	16.38
	Cluster-4820.33818	0.12	0.25	0.59	2.25	1.16	1.43
	Cluster-4820.4585	1.96	0.61	0.76	6.22	7.39	8.68
	Cluster-4820.79967	0.75	0	0.55	82.09	68.29	88.57
